# Loss of Visual Acuity after Successful Surgery for Macula-On Rhegmatogenous Retinal Detachment in a Prospective Multicentre Study

**DOI:** 10.1155/2015/821864

**Published:** 2015-11-12

**Authors:** Salvatore Di Lauro, Melissa Castrejón, Itziar Fernández, Jimena Rojas, Rosa M. Coco, María R. Sanabria, Enrique Rodríguez de la Rua, J. Carlos Pastor

**Affiliations:** ^1^IOBA (Eye Institute), University of Valladolid, 47011 Valladolid, Spain; ^2^Hospital Clínico Universitario de Valladolid, 47003 Valladolid, Spain; ^3^Ciber-BBN, 47011 Valladolid, Spain; ^4^Complejo Asistencial de Palencia (CAPA), 34005 Palencia, Spain; ^5^Hospital Universitario Virgen Macarena y Hospital Universitario Virgen del Rocío, 41009 Sevilla, Spain

## Abstract

*Purpose*. To quantify the frequency of visual loss after successful retinal detachment (RD) surgery in macula-on patients in a multicentric, prospective series of RD.* Methods*. Clinical variables from consecutive macula-on RD patients were collected in a prospective multicentric study. Visual loss was defined as at least a reduction in one line in best corrected visual acuity (VA) with Snellen chart. The series were divided into 4 subgroups: (1) all macula-on eyes (*n* = 357); (2) macula-on patients with visual loss at the third month of follow-up (*n* = 53) which were further subdivided in (3) phakic eyes (*n* = 39); and (4) pseudophakic eyes (*n* = 14).* Results*. Fifty-three eyes (14.9%) had visual loss three months after surgery (*n* = 39 phakic eyes; *n* = 14 pseudophakic eyes). There were no statistically significant differences between them regarding their clinical characteristics. Pars plana vitrectomy (PPV) was used in 67.2% of cases, scleral buckle in 57.7%, and scleral explant in 11.9% (36.1% were combined procedures).* Conclusions*. Around 15% of macula-on RD eyes lose VA after successful surgery. Development of cataracts may be one cause in phakic eyes, but vision loss in pseudophakic eyes could have other explanations such as the effect of released factors produced by retinal ischemia on the macula area. Further investigations are necessary to elucidate this hypothesis.

## 1. Introduction

Macula-on rhegmatogenous retinal detachment (RD) is a common cause of ophthalmic emergency that requires surgical treatment in a short period of time to prevent visual loss [1]. The annual incidence of RD varies according to the recent references between 15.4 [2] and 18.2 per 100 000, with a peak incidence of 52.5 per 100 000 people between 55 and 59 years of age [3]. Myopia is a major risk factor, with a 4-fold higher risk of RD, compared with a nonmyopic eye, in eyes with mild myopia and a 10-fold higher risk in eyes with moderate and high myopia (greater than −3 diopters) [4]. The risk of RD is 4 times higher in patients who have undergone cataract surgery [5, 6].

The repair of RD has improved during the last 20 years, and today the goal is not only to reattach the retina but to achieve an adequate final vision. The development of better surgical techniques, such as scleral buckling, pneumatic retinopexy, and pars plana vitrectomy (PPV), has continued to improve the anatomical success rates, going up from 70% to 90% and achieving better functional outcomes [7–9]. Nevertheless, proper end vision recovery is a challenge in many cases of RD [10]. Besides adequate anatomical reattachment, other clinical factors have been identified as determinants that prevent a good functional recovery. The status of the macula is the main factor for a successful functional result [10–12]. Even in a successful RD surgery with a clinically normal macular area, the final vision may be subnormal. Poor visual acuity (VA), color vision defects, and metamorphopsia have been described in successful postoperative RD macula-on surgeries, suggesting the existence of microstructural retina damage [12].

Many series of cases have been reported with excellent anatomical results but not so for the functional outcome after RD surgery. In fact, many surgeons assume that having macula-on RD will always have a better visual function prognosis than those with macula-off. And there are few papers dealing with this topic. We performed a PubMed search for English language manuscripts reporting RD and macula-on functional outcomes published since 2003, and only 4 reports were identified [10, 11, 13, 14]. For this reason we have evaluated data collected in a prospective multicentric study including 17 centers in Spain and Portugal, with data from RDs consecutively treated which was primarily designed to add more information on PVR development and clinical risk factors [15–19]. The purpose of this report is to analyze those macula-on RD cases that were anatomical successful treated but experienced loss of vision at the third month of follow-up.

## 2. Methods

### 2.1. Patient Population

Seventeen clinical ophthalmological institutions in Spain and Portugal participated in the study [12–16]. As mentioned the project was designed to improve the sensitivity and specificity of a formula published by Rodríguez De La Rúa et al. [20] by using clinical factors for calculating the risk of developing proliferative vitreoretinopathy (PVR) after RD surgery. A total of 1.047 consecutive RD cases fulfilled the inclusion criteria. For that project, a database record was created collecting 83 variables that included all pre-, intra-, and postoperative characteristics [20].

The study protocol was approved by the research committee of the coordinating institution (IOBA, University of Valladolid, Spain) and by each of the participating centers. Informed consent was obtained from each patient before inclusion of his/her data in a common database. This research followed the tenets of the Declaration of Helsinki.

### 2.2. Inclusion and Exclusion Criteria

All patients consecutively admitted for surgery between October 2004 and February 2008 with RD and a minimum follow-up of three months were considered for inclusion [19]. For this present analysis only patients with a macula-on RD at the time of the surgery and the macula-on during the entire follow-up were included. Cases of preoperative PVR grade C-1 or higher according to the Retina Society Classification [21] and posttraumatic RD were excluded. Patients without completely reattached retina at the end of follow-up were excluded. Patients with incomplete data regarding the status of the macula were also excluded from the final analysis.

During the period of time when these patients were included, not all the centers had optical coherent tomography (OCT) technology available, and it was not considered as a routine component of the RD patient examination. Therefore, the status of the macula was determined by indirect ophthalmoscopy and posterior biomicroscopy in the immediate preoperative period (less than 24 hours before surgery).

From the RD patients included in the whole study, 357 had macula-on RDs, and they were considered the subject sample of this study. The patients were divided into 4 groups for further analysis according to the status of the lens and the functional outcome after 3 months of follow-up. A reduction of at least one line of best corrected VA (BCVA) in the Snellen chart at the third month of follow-up was considered as visual loss. Group 1 (G1, *n* = 357) included the whole sample of macula-on patients. Group 2 (G2, *n* = 53) included macula-on patients with reattached retina and who experienced visual loss at the third month of follow-up. Group 3 (G3, *n* = 39) included phakic eyes out of G2, and Group 4 (G4, *n* = 14) involved the pseudophakic eyes out of G2.

The main preoperative characteristics recorded for G2, G3, and G4 were retinal breaks type (tear, hole, dialysis, and giant tear), size of retinal breaks in clock hours, extension of RD in quadrants, presence of preoperative PVR less than grade C, previous aphakia, previous vitreous hemorrhage, posterior vitreous detachment, myopia, and preoperative visual acuity (Table 1).

### 2.3. Variables

Only 27 out of the total 83 characteristics gathered in the whole study [20] were used for the analysis of this report. Surgeons participating in the study were highly experienced in RD treatment and were allowed to treat their patients on their own criteria and according to their personal experience. Reinterventions were permitted. The endotamponade with PPV was diverse, including none, air, SF6, C3F8, and silicone oil. Retinopexy was achieved with cryopexia or endolaser. Most of the technical details of the whole series have been already published [15–20].

Anatomical success was defined as a reattached retina at the last postoperative follow-up at three months. Functional outcome was recorded as BCVA with the Snellen chart at the initial (preoperative) visit and at the final 3-month follow-up visit. For statistical analysis, BCVA data were grouped into three ranges: >20/40, 20/50 to 20/100, and <20/100.

### 2.4. Statistical Analysis

Qualitative variables were described by percentages and quantitative ones by mean ± standard deviation (SD). In all cases, 95% confidence intervals (95%CI) were calculated. Groups were compared by difference in proportions tests. Contingency tables were constructed to evaluate the association between categorical RD characteristics and anatomical and functional outcomes. The independence was checked by Chi-square test or Fisher's exact test on sparse tables. Statistical significance was established at the 0.05 confidence level. Statistical analysis was conducted with SPSS (v.19, SPSS, Chicago, IL, USA) and R software [22].

## 3. Results

The average age of the G1 RD subjects was 52.9 ± 15.6 years when the surgery was performed. The mean time between onset of RD signs or symptoms and surgery was 13.3 ± 24.9 days, although all patients were macula-on at the time of surgery. Comparing the preoperative characteristics of the overall study patients, the G1 patients, all of whom were in the macula-on group, were statistically different for the following (Table 2). (1) Absence of clinical signs of PVR at the preoperative examination was more frequent in G1. (2) RD with an extension of 1 quadrant was more frequent in G1. (3) Percentage of previous cataract surgery in G1 is slightly higher. (4) Eyes without myopia were less frequent in G1. And (5) preoperative VA was significantly higher in G1. Differences for the other pre-, intra-, and postoperative clinical characteristics were not statistically significant. The main preoperative characteristics in G1 compared to the whole study series cohort are shown in Table 2. In G1, 345 eyes (96.6%; 95%CI: 94–98.2%) had reattached retinas at the end of follow-up. In 12 eyes, the central retina was not reattached; therefore, they were excluded from further analysis. Single reattachment procedures included PPV (67.2%; 95%CI: 62.1–72%), scleral buckle (57.7%, 95%CI: 52.4%; 62.9%), and explant (11.9%; 95%CI: 8.8–15.9%). Combined procedures were performed on 36.1% (95%CI: 31.2–41.4%) of G1.

The mean BCVA in G1 was 0.57 ± 0.3 in the preoperative visit and 0.59 ± 0.29 at 3 months of follow-up. The preoperative VA in G1 was <20/100 in 44 patients (12.3%; 95%CI: 8.9%, 15.7%), between 20/100 and 20/40 in 79 eyes (22.1%; 95%CI: 17.8%, 26.4%), and >20/40 in 234 eyes (65.6%; 95%CI: 60.6%, 70.5%) (Table 2). The postoperative VA in this group at the end of the follow-up was <20/100 in 37 eyes (10.4%; 95%CI: 7.2%, 13.5%), between 20/100 and 20/40 in 80 eyes (22.4%; 95%CI: 18.1%, 26.7%), and >20/40 in 240 eyes (67.2%; 95%CI: 62.4%, 72.1%). Twenty-six out of 37 eyes with a final VA <20/100 (7.3%; 95%CI: 4.9%, 10.6%) had a decreased VA at the end of the follow-up.

In G2 (Table 1), which included phakic and pseudophakic eyes with reattached retinas and visual loss, the final VA, 0.23 ± 0.14, was lower than the initial VA, 0.65 ± 0.25 (*p* < 0.0001, paired *t*-test), despite reattachment of the retina. The average age of these patients was 18.5 ± 28.7 days. PPV was the most commonly performed surgery (88.7%; 95%CI: 80.2–97.2%) followed by scleral buckle surgery (58.5%; 95%CI: 45.2–71.8%). In some cases, both procedures were used.

In G3 (Tables 1 and 3), which included 39 phakic eyes with reattached retinas but with a reduction in VA at the end of follow-up, the average age was 54.1 ± 16.5 years at the onset of RD. The mean time between onset of RD and surgery was 17.7 ± 24.9 days. In the preoperative VA evaluation, 82.1% of eyes (*n* = 32) had >20/40 and none of them had <20/100 but at the end of the third month 30.8% (*n* = 12) had <20/100. The main preoperative characteristics of this group were the following: (1) no PVR was present in 48.7%, whereas PVR grades A and B were present in 33.3% and 17.9%, respectively, (2) a visible retinal tear and a unique break were present in 97.4% and 52.6%, respectively, (3) RD extensions of 2 to 3 quadrants were found in 59% and complete posterior vitreous detachment (PVD) was present in 53,8% of eyes, and (4) PPV was performed on 87.2% (95%CI: 71.8–95.2%) of the patients and scleral buckle surgery on 64.1% (95%CI: 47.2–78.3%). Some patients received both procedures.

In G4 (Tables 1 and 4), which comprised 14 pseudophakic patients with macula-on and successful surgery but who had visual loss at the end of the follow-up, the average age was 63.3 ± 14.4 years. The mean time between onset of RD and surgery was 20.7 ± 38 days. The initial VA was >20/40 in 12 eyes (85.7%). At the end of the follow-up, the VA was <20/100 in 50% and between 20/100 and 20/40 in 50% of the cases (Table 4). PPV was performed on 92.9% (95%CI: 85.9%; 99.8%) of the patients and scleral buckle surgery on 42.9% (95%CI: 29.5; 56.2%) (*p* < 0.05). As in the other groups, some patients received both procedures.

## 4. Discussion

While there are published data regarding the improvement of anatomical results of RD surgery, there are few reports showing whether or not RD surgery results in functional improvement [9–11, 13, 14, 23, 24] and, to the best of our knowledge, there is only a report regarding visual loss in macula-on RD after successful surgery [10]. For RD certain variables are thought to be significant predictors for functional outcome, for instance, the status of the macula (which is the most important factor) [25], macular elevation when detached [26], and other factors such as age of the patient [9, 25], poor initial vision [9, 25], and interval between onset of RD and surgery [23, 27–29]. It is generally assumed by most surgeons that the “macula-on” status before surgery guarantees good functional results, which in most cases means to preserve the patients previous VA. However, we have experienced macula-on RD cases in which despite successful surgery the visual results were not favorable. This experience is common among the retinal surgeons but as mentioned there is no specific publication on this topic.

Thus, although the original study was not specifically set up to investigate this event, we used its data to analyze the frequency of unfavorable outcomes and to add some clinical information.

In this study, 53 out of 357 patients (14.9%; 95%CI: 11.4–19.1%) meeting the inclusion criteria experienced a worsening of VA after RD surgery although the macula was attached. The American Academy of Ophthalmology has recognized that the status of the macula is the major factor related to visual prognosis [1]. It recommends that when the RD does not affect the macula, repair should be made within the first 24 hours following the diagnosis [1]. In our series, the mean time between the onset of the symptoms and surgery was 13.3 ± 24.9 days. Nevertheless, the surgery was always performed within 72 hours after the admittance at the hospital, although symptoms started several days before. Because the central vision was not affected by the RD, it is likely that both the patients and the general practitioners underestimated the severity of the disease and that may explain the delayed diagnosis. In addition, photopsias could appear several days or even weeks before the development of RD. Then, long period of symptoms does not necessarily indicate a long standing RD. This long interval before surgery was already mentioned by our group and could be a fact related to the structure of the National Health System, where most of the patients complaining floaters or flashes are referred to the general practitioner instead of ophthalmologist (GP) [16, 30]. It is acknowledged that the time interval to surgery is crucial to prevent progression of subretinal fluid into the macula and disruption of the photoreceptors, causing a possible loss of VA in some cases [31]. For our patients with macula-on RDs, the evaluations were made only by indirect ophthalmoscopy or fundus biomicroscopy at 24 or 48 hours prior to the surgery. Thus, it is possible that we could not detect the presence of a small amount of subretinal fluid under the macula as a limitation of this study.

It is possible that systematic analysis of the status of the macula by OCT may increase the number of macula-off patients and also that structural changes in patients with visual loss could be detected. Recent studies used Fourier-domain OCT to evaluate postoperatively both macula-on and macula-off RDs [32, 33]. Foveal anatomical abnormalities were discovered in 62% of the patients. In macula-off cases, there was disruption of the photoreceptor inner segment/outer segment (IS/OS) junction line (which correspond to the ellipsoid portion of the photoreceptors: ellipsoid) in 61% of the cases, and the external limiting membrane (ELM) was observed only in 24%. Additional changes were noted in both types of detachments. These disruptions were statistically related to relatively poor postoperative VA. When followed up overtime, the restoration of a normal IS/OS line occurred only in eyes without an initially disrupted ELM as the investigators also noted. Eyes in which the IS/OS junction returned to normal showed better VA than those in which this change did not take place [31]. It is important to take into consideration, however, that although OCT is currently a widespread tool, it could take time until patient performs it. Considering the timing for the management of this kind of RD, OCT become sometimes indispensable auxiliary test.

As mentioned we did not perform OCT on patients preoperatively to rule out a subclinical extension of subretinal fluid under the fovea, a reason which could explain why some eyes with similar alterations have functional results significantly better than others [34].

We analyzed phakic and pseudophakic groups separately for avoiding the bias of cataract progression. For phakic eyes that experienced loss of vision in spite of successful surgery, especially in those operated by vitrectomy, it is possible that the low vision could have been caused by the progression of cataracts [35]. However, there were 14 pseudophakic eyes in the G4 group that experienced visual loss that could not be attributed to progression of lens opacity. Therefore, other factors must be implicated. Some factors such as intraoperative complications like rise intraocular pressure (IOP) and microscope light toxicity during the surgery, as well as the use of silicon oil or gas as tamponade, could have produced retinal damage, causing a reduction in the inner retinal thickness due to neuronal cell loss in the macular area [36]. However, no postsurgical rise in IOP was detected in this series and no one received silicon oil tamponade in these groups, so these variables can be safely excluded as the cause of visual loss in our patients. Neither epiretinal membranes nor cystoid macular edema, which are also causes of visual loss after RD surgery, was identified in the follow-up. When these conditions were suspected most of the patients were evaluated by OCT, when possible. As no clinical differences were found between groups, further investigations should elucidate if causes of visual loss are related to a gross anatomic factor or could be a photoreceptor damage caused by diffusible factors released by ischemic detached retina [37]. In fact oxidative damage and TNF-*α* are factors used experimentally for inducing damage to the RPE cells and photoreceptors [38]. Finally, analysis of retinas after experimental retinal detachments in cats reveals that ultrastructural changes (apoptosis, gliosis) occur beyond the specific area of the detached retina [39]. These changes could be one the reasons of the impairment of vision in our patients.

This study has some important limitations. First is that the study design was not intended to solve the question of vision loss in patients with macula-on RD, but, in view of the lack of reports on VA loss after macula-on RD surgery, we have considered that our data could be interesting. Second limitation is that at the time of inclusion of patients OCT was not available in all centers, and those having this technology had no similar equipment. Finally the study was restricted to a specific area of Spain and Portugal. So there is no way to know for sure if the data may be extrapolated to other geographical areas, although our Retina 1 Project reports have yielded similar results of RD surgery to those obtained by other neighboring countries [9, 12, 35, 40].

## 5. Conclusions

In summary, we have added information on the prevalence of the clinically significant loss of VA following RD with macula-on surgery, a condition affecting a relevant percentage of patients. Neither clinical nor surgical factors were identified as responsible. This topic deserves further study, incorporating new technical devices such as swept source OCT that is able to identify structural changes at the level of the inner retina. Biochemical studies could also elucidate in the future whether or not any molecule released by the detached retina may be responsible for this damage.

## Figures and Tables

**Table 1:** Main preoperative characteristics in groups G2, G3, and G4.

	Eyes with reattached retina and visual loss (G2)			Phakic eyes with reattached retina and visual loss (G3)			Pseudophakic eyes with reattached retina and visual loss (G4)		
	*n* (%)	95%CI	*p* value^*∗*^	*n* (%)	95%CI	*p* value^*∗*^	*n* (%)	95%CI	*p* value^*∗*^
Previous PVR									
No	24 (45.3%)	31.9%; 58.7%	0.1166	19 (48.7%)	32.7%; 65%	0.3842	5 (35.7%)	22.8%; 48.6%	0.1832
A	17 (32.1%)	19.5%; 44.6%	0.2695	13 (33.3%)	19.6%; 50.3%	0.2799	4 (28.6%)	16.4%; 40.7%	0.9723
B	12 (22.6%)	11.4%; 34%	0.5488	7 (17.9%)	8.1%; 34.1%	1	5 (35.7%)	22.8%; 48.6%	0.1886
Total	53 (100%)			39 (100%)			14 (100%)		
Retinal break									
Tear	38 (71.7%)	59.6%; 83.8%	0.9803	28 (71.8%)	54.9%; 84.5%	1	10 (71.4%)	59.3%; 83.6%	1
Hole	12 (22.6%)	11.4%; 33.9%	0.8577	9 (23.1%)	11.7%; 39.7%	0.866	3 (21.4%)	10.4%; 32.5%	1
Dialysis	0 (0%)	0%; 8.4%	0.61	0 (0%)	0%; 11.2%	0.7774	0 (0%)	0%; 26.8%	1
Giant tear	2 (3.8%)	0%; 8.9%	1	1 (2.6%)	0.1%; 15.1%	0.9861	1 (7.1%)	0.2%; 14.1%	1
Not visible	1 (1.9%)	0%; 5.6%	1	1 (2.6%)	0.1%; 15.1%	1	0 (0%)	0%; 26.8%	1
Total	53 (100%)			39 (100%)			14 (100%)		
Type of break									
Single	27 (52.9%)	39.2%; 66.6%	0.7103	20 (52.6%)	36.1%; 68.7%	0.7509	7 (53.9%)	40.2%; 67.5%	1
Multiple	22 (43.1%)	29.5%; 56.7%	0.6979	16 (42.1%)	26.7%; 59.1%	0.8708	6 (46.2%)	32.5%; 59.8%	0.8359
Posterior rupture	1 (2%)	0%; 5.8%	1	1 (2.6%)	0.1%; 15.4%	0.9477	0 (0%)	0%; 28.3%	1
Not visible	1 (2%)	0%; 5.8%	1	1 (2.6%)	0.1%; 15.4%	1	0 (0%)	0%; 28.3%	1
Total	51 (100%)			38 (100%)			13 (100%)		
Size of breaks (clock hours)									
Not visible	1 (1.9%)	0%; 5.7%	0.9947	1 (2.6%)	0.1%; 15.4%	1	0 (0%)	0%; 26.8%	1
0-1	31 (59.6%)	46.3%; 73%	0.3571	22 (57.9%)	40.9%; 73.3%	0.3341	9 (64.3%)	51.3%; 77.3%	1
2-3	14 (26.9%)	14.9%; 39%	0.6371	11 (28.9%)	16%; 46.1%	0.5175	3 (21.4%)	10.3%; 32.6%	1
>3	6 (11.5%)	2.9%; 20.2%	0.3815	4 (10.5%)	3.4%; 25.7%	0.6698	2 (14.3%)	4.8%; 23.8%	0.6357
Total	52 (100%)			38 (100%)			14 (100%)		
Previous vitreous hemorrhage									
No	44 (83%)	72.9%; 93.1%	0.6852	32 (82.1%)	65.9%; 91.9%	0.6451	12 (85.7%)	76.3%; 95.1%	1
Yes	9 (17%)	6.9%; 27.1%	0.6852	7 (17.9%)	8.1%; 34.1%	0.6451	2 (14.3%)	4.9%; 23.7%	1
Total	53 (100%)			39 (100%)			14 (100%)		
Extension RD (quadrants)									
0-1	21 (39.6%)	26.5%; 52.8%	0.9019	16 (41%)	26%; 57.8%	1	5 (35.7%)	22.8%; 48.6%	0.8724
2-3	32 (60.4%)	47.2%; 73.6%	0.9019	23 (59%)	42.2%; 74%	1	9 (64.3%)	51.4%; 77.2%	0.8724
4	0 (0%)	0%; 8.4%	1	0 (0%)	0%; 11.2%	1	0 (0%)	0%; 26.8%	1
Total	53 (100%)			39 (100%)			14 (100%)		
Previous aphakic									
No	39 (73.6%)	61.7%; 85.5%	0.6412	39 (100%)	88.8%; 100%	0.0001	0 (0%)	0%; 26.8%	<0.0001
Yes	14 (26.4%)	14.6%; 38.3%	0.6412	0 (0%)	0%; 11.2%	0.0001	14 (100%)	73.2%; 100%	<0.0001
Total	53 (100%)			39 (100%)			14 (100%)		
PVD									
Unknown	11 (20.8%)	9.8%; 31.7%	0.1176	8 (20.5%)	9.9%; 36.9%	0.1837	3 (21.4%)	10.4%; 32.5%	0.59
No	13 (24.5%)	12.9%; 36.1%	0.8361	10 (25.6%)	13.6%; 42.4%	0.7692	3 (21.4%)	10.4%; 32.5%	1
Yes	29 (54.7%)	41.3%; 68.1%	0.2562	21 (53.8%)	37.4%; 69.6%	0.4108	8 (57.1%)	43.8%; 70.5%	0.5698
Total	53 (100%)			39 (100%)			14 (100%)		
Myopia									
No	11 (27.5%)	13.7%; 41.3%	0.5078	9 (29%)	14.9%; 48.2%	0.719	2 (22.2%)	9.3%; 35.1%	0.707
≤5 Dp	15 (37.5%)	22.5%; 52.5%	0.662	12 (38.7%)	22.4%; 57.7%	0.6276	3 (33.3%)	18.7%; 47.9%	1
>5 Dp	14 (35%)	20.2%; 49.8%	0.9518	10 (32.3%)	17.3%; 51.5%	1	4 (44.4%)	29%; 59.8%	0.722
Total	40 (100%)			31 (100%)			9 (100%)		
Previous VA									
<20/100	0 (0%)	0%; 8.4%	0.0118	0 (0%)	0%; 11.2%	0.0362	0 (0%)	0%; 26.8%	0.32
20/100–20/40	9 (17%)	6.9%; 27.1%	0.4638	7 (17.9%)	8.1%; 34.1%	0.6659	2 (14.3%)	4.9%; 23.7%	0.702
≥20/40	44 (83%)	72.9%; 93.1%	0.0111	32 (82.1%)	65.9%; 91.9%	0.0449	12 (85.7%)	76.3%; 95.1%	0.1902
Total	53 (100%)			39 (100%)			14 (100%)		

^*∗*^Differences from G1: macula-on series.

*n* (%): number and percentage of patients in Retina 1 Project and macula-on groups G1, G2, G3, and G4. G1: macula-on group, G2: phakic and pseudophakic patients, G3: phakic patients, and G4: pseudophakic patients; CI: confidence interval; PVR: proliferative vitreoretinopathy; PVD: posterior vitreous detachment. VA: visual acuity, best corrected visual acuity.

**Table 2:** Mean preoperative characteristics of the Retina 1 series included in the G1 group.

	Whole series (Retina 1)	Macula-on group (G1)		*p* value
	*n* (%)	*n* (%)	95%CI	
Previous PVR				
No	522 (49.9%)	203 (56.9%)	51.7%; 62%	0.0095
A	247 (23.6%)	88 (24.7%)	20.2%; 29.1%	0.6823
B	278 (26.6%)	66 (18.5%)	14.5%; 22.5%	0.0007
Total	1047 (100%)	357 (100%)		
Retinal break				
Tear	739 (70.7%)	252 (70.6%)	65.9%; 75.3%	1
Hole	223 (21.3%)	74 (20.7%)	16.5%; 24.9%	0.8278
Dialysis	12 (1.2%)	7 (2%)	0.5%; 3.4%	0.2346
Giant tear	37 (3.5%)	14 (3.9%)	1.9%; 5.9%	0.805
Not visible	34 (3.3%)	10 (2.8%)	1.1%; 4.5%	0.7421
Total	1045 (100%)	357 (100%)		
Type of break				
Single	526 (52.1%)	196 (56.5%)	51.3%; 61.7%	0.1164
Multiple	432 (42.8%)	137 (39.5%)	34.3%; 44.6%	0.2306
Posterior rupture	17 (1.7%)	4 (1.2%)	0%; 2.3%	0.5786
Not visible	34 (3.4%)	10 (2.9%)	1.1%; 4.6%	0.7225
Total	1009 (100%)	357 (100%)		
Size of breaks (clock hours)				
Not visible	34 (3.3%)	10 (2.9%)	1.1%; 4.6%	0.7321
0-1	673 (65.4%)	235 (66.6%)	61.7%; 71.5%	0.684
2-3	265 (25.8%)	82 (23.2%)	18.8%; 27.6%	0.3067
>3	57 (5.5%)	26 (7.4%)	4.6%; 10.1%	0.1667
Total	1029 (100%)	353 (100%)		
Previous vitreous hemorrhage				
No	908 (88%)	304 (85.9%)	82.3%; 89.5%	0.2561
Yes	124 (12%)	50 (14.1%)	10.5%; 17.8%	0.2561
Total	1032 (100%)	354 (100%)		
Extension RD (quadrants)				
0-1	188 (18.1%)	147 (41.4%)	36.3%; 46.5%	<0.0001
2-3	736 (70.8%)	208 (58.6%)	53.5%; 63.7%	<0.0001
4	116 (11.2%)	0 (0%)	0%; 1.3%	<0.0001
Total	1040 (100%)	355 (100%)		
Macula				
On	382 (36.7%)	357 (100%)	98.7%; 100%	<0.0001
Off	658 (63.3%)	0 (0%)	0%; 1.3%	<0.0001
Total	1040 (100%)	357 (100%)		
Previous aphakic				
No	664 (63.5%)	248 (69.7%)	64.9%; 74.4%	0.0179
Yes	382 (36.5%)	108 (30.3%)	25.6%; 35.1%	0.0179
Total	1046 (100%)	356 (100%)		
PVD				
Unknown	357 (34.1%)	113 (31.7%)	26.8%; 36.5%	0.3578
No	193 (18.4%)	80 (22.4%)	18.1%; 26.7%	0.0614
Yes	497 (47.5%)	164 (46%)	40.8%; 51.1%	0.5985
Total	1047 (100%)	357 (100%)		
Myopia				
No	346 (44.5%)	94 (33.7%)	28.1%; 39.2%	0.0003
≤5 Dp	206 (26.5%)	92 (33%)	27.5%; 38.5%	0.0174
>5 Dp	225 (29%)	93 (33.3%)	27.8%; 38.9%	0.1225
Total	777 (100%)	279 (100%)		
Preoperative VA				
<20/100	631 (61%)	44 (12.3%)	8.9%; 15.7%	<0.0001
20/100–20/40	142 (13.7%)	79 (22.1%)	17.8%; 26.4%	<0.0001
≥20/40	261 (25.2%)	234 (65.6%)	60.6%; 70.5%	<0.0001
Total	1034 (100%)	357 (100%)		

*n* (%): number and percentage of patients in Retina 1 Project and macula-on group (G1); G1: macula-on group; CI: confidence Interval; PVR: proliferative vitreoretinopathy; PVR grades A and B as defined by the Retina Society Classification in 1983; PVD: posterior vitreous detachment; RD: retinal detachment; VA: visual acuity, best corrected visual acuity.

**Table 3:** Variation in visual acuity in G3 before surgery and at the end of the follow-up period.

VA	Preoperative VA		Postoperative VA	
	*n* (%)	95%CI	*n* (%)	95%CI
<20/100	0 (0%)	0%; 11.2%	12 (30.8%)	17.6%; 47.7%
20/100–20/40	7 (17.9%)	8.1%; 34.1%	27 (69.2%)	52.3%; 82.5%
>20/40	32 (82.1%)	65.9%, 91.9%	0 (0%)	0%; 11.2%
Total	39 (100%)		39 (100%)	

G3: phakic eyes with visual loss at third month of follow-up; VA: best visual acuity corrected at the end of the follow-up (Snellen chart).

**Table 4:** Variation in visual acuity in G4 before surgery and at the end of the follow-up.

VA	Preoperative VA		Postoperative VA	
	*n* (%)	95%CI	*n* (%)	95%CI
<20/100	0 (0%)	0%; 26.8%	7 (50%)	36.5%; 63.5%
20/100–20/40	2 (14.3%)	4.9%; 23.7%	7 (50%)	36.5%; 63.5%
>20/40	12 (85.7%)	76.3%; 95.1%	0 (0%)	0%; 26.8%
Total	14 (100%)		14 (100%)	

G4: pseudophakic eyes with visual loss at third month of follow-up; VA: best visual acuity corrected at the end of the follow-up (Snellen chart).
